# Unraveling the intersection of aging and Parkinson’s disease: a collaborative roadmap for advancing research models

**DOI:** 10.1038/s41531-025-01239-x

**Published:** 2026-01-16

**Authors:** M. Y. Schmidt, A. M. Cuervo, K. L. Double, D. Ehninger, M. S. Goldberg, K. Harvey, J. H. J. Hoeijmakers, K. C. Luk, P. G. Mastroberardino, D. J. Moore, L. J. Niedernhofer, L. É. Trudeau, D. Jurk, J. K. Andersen, I. Bellantuono

**Affiliations:** 1 Buck Institute for Research on Aging, Novato, CA USA; 2 Institute for Aging Research, Albert Einstein College of Medicine, New York, NY USA; 3 Department of Developmental and Molecular Biology, Albert Einstein College of Medicine, New York, NY USA; 4 Brain and Mind Centre and School of Medical Sciences (Neuroscience), Faculty of Medicine and Health, The University of Sydney, Sydney, NSW Australia; 5 Translational Biogerontology Lab, German Center for Neurodegenerative Diseases (DZNE), Bonn, Germany; 6 Department of Neurology, Center for Neurodegeneration and Experimental Therapeutics, University of Alabama at Birmingham, Birmingham, AL USA; 7 Department of Pharmacology, UCL School of Pharmacy, University College London, London, UK; 8 Department of Molecular Genetics, Erasmus MC Cancer Institute, Erasmus University Medical Center, Rotterdam, The Netherlands; 9 University Hospital of Cologne, CECAD Forschungszentrum, Institute for Genome Stability in Aging and Disease, Köln, Germany; 10 Princess Maxima Center for Pediatric Oncology, Oncode Institute, Utrecht, The Netherlands; 11 Department of Pathology and Laboratory Medicine, Center for Neurodegenerative Disease Research, Perelman School of Medicine, University of Pennsylvania, Philadelphia, PA USA; 12 IFOM-ETS, The AIRC Institute for Molecular Oncology, Milan, Italy; 13 Università degli Studi dell’Aquila, L’Aquila, Italy; 14 Department of Neurodegenerative Science, Van Andel Institute, Grand Rapids, MI USA; 15 Institute on the Biology of Aging and Metabolism, Department of Biochemistry, Molecular Biology and Biophysics, University of Minnesota, Minneapolis, MN USA; 16 Department of Pharmacology and Physiology, Department of Neurosciences, Faculty of Medicine, Université de Montréal, Montréal, QC Canada; 17 Neural Signalling and Circuitry Research Group (SNC), Center for Interdisciplinary Research on the Brain and Learning (CIRCA), Center for Biomedical Innovation (CIB), Université de Montréal, Montréal, QC Canada; 18 Mayo Clinic, Department of Physiology and Biomedical Engineering, Robert and Arlene Kogod Center on Aging, Rochester, MN USA; 19 School of Medicine and Population Health, Healthy Lifespan Institute, University of Sheffield, Sheffield, UK

**Keywords:** Parkinson's disease, Neuroscience, Risk factors

## Abstract

Aging is the most significant risk factor for Parkinson’s disease (PD), yet its role remains underexplored. The International Network for Parkinson’s Disease Modelling and Aging (PD-AGE), funded by the Michael J. Fox Foundation, was established to address these challenges. Through collaborative efforts, the PD-AGE mouse-model working group developed a roadmap to prioritize mouse models, reach consensus on experimental approaches, and standardize protocols to investigate the intersection of aging and PD.

## Introduction

Mouse models of Parkinson’s disease (PD) are invaluable for advancing our understanding of the disease, and there is much hope that their use will help develop new therapeutic interventions. PD is a complex multisystem disorder characterized by a spectrum of motor and non-motor symptoms, and numerous mouse models have been developed to study its various aspects. While age is the primary risk factor for PD, the role of biological aging in PD is still unclear, and it is often overlooked in the design and application of these models. This omission risks missing critical insights into disease mechanisms and opportunities for the development and translation of novel interventions, in particular as aging biology is emerging as a therapeutic target^1^.

The International Network for Parkinson’s Disease Modelling and AGEing (PD-AGE), funded by the Michael J. Fox Foundation for Parkinson’s Research, was established to address critical gaps in our understanding of the role of aging in PD. Its creation was prompted by a workshop that brought together leading experts in PD modeling and aging who collectively highlighted the need for a systematic investigation into how aging contributes to PD^2^. Insights into the mechanisms linking aging to PD remain limited and contradictory, making this research direction a high priority. However, the complexity of PD, evidenced by the heterogeneity of patient phenotypes and the diversity of available preclinical models, along with the complexity of the aging process, which involves multiple hallmarks and unfolds heterogeneously, poses significant challenges that are more effectively addressed through a collaborative approach 1.

To achieve its goals, PD-AGE was divided into four working groups, each focusing on different models. Here, we report on the working group that focused on approaches using mouse models and conducted a series of workshops to build consensus on prioritizing models of aging and PD, experimental approaches, and the standardization of protocols for their characterization. The result is a comprehensive roadmap for selecting optimal models, defining relevant measurements, and harmonizing protocols. This roadmap establishes a foundation for coordinated efforts, fostering resource sharing, knowledge exchange, and data integration to accelerate progress in understanding the intersection of aging and PD.

## Parkinson’s disease and aging

PD is considered an age-related progressive neurodegenerative syndrome, which can be grouped into four general stages—prodromal, early, intermediate, and late.

Several observations suggest that biological aging may actively shape disease trajectories, contributing to the interindividual variability observed in PD symptom severity and progression. The prevalence of PD has risen markedly in recent decades due in part to aging populations in many countries^3^. Advancing age is the most significant risk factor for PD as evidenced by its markedly higher prevalence in older populations. The incidence rate increases from 41 per 100,000 among individuals aged 40–49, to 1903 per 100,000 in those over 80 years old in the United States^4^. Similar trends (per 100,000) are reported in low and middle-income countries: 7 for people between 40 and 49 years old, 158 for 50–59 years old, 603 for 60–69 years old, 1251 for 70–79 years old, and 2181 in those over the age of 80^5^. In Europe, the overall prevalence in persons 65 years of age and older (per 100,000) was 1800, with an increase from 600 for those aged 65–69 years, to 2600 for those 85–89 years^6^. While genetic factors likely play an etiological role, most cases of PD are sporadic, with only 10% of PD patients presenting a known family history^7^. Mutations in genes such as *SNCA* (α-synuclein), *Pink1* (Pten-induced kinase 1), and *PRKN* (Parkin) are predominantly linked to early-onset PD, whereas mutations in other genes, including *LRRK2* (Leucine-Rich Repeat Kinase 2) and *GBA* (glucocerebrosidase), are recognized contributors to mid and late-onset PD, respectively. In contrast, a mix of factors, including age, genetic vulnerability, environmental exposures, and poor lifestyle (e.g., diet and exercise), are associated with increased risk of disease in sporadic forms of PD^8–10^. Importantly, these same factors influence the rate of biological aging^11–13^ — system-level changes in cellular and molecular processes, such as mitochondrial function, proteostasis, genomic stability, inflammation, and senescence, accumulate across lifespan and increase vulnerability to disease. Such factors can accelerate the rate of biological aging depending on the individual’s exposures. Furthermore, their impact can vary substantially between individuals, as well as within the tissues of the same individual^14^, generating a high level of heterogeneity for driving disease onset and progression. This could potentially offer an explanation for the heterogeneity also observed in PD, which is evident in the presentation of symptomatology, age of onset, and rate of progression.

Importantly, many age-related changes in the brain mirror those observed in the early stages of PD^2^. Furthermore, many of the hallmarks of aging, including mitochondrial dysfunction, impaired autophagy, increased inflammation, and cellular senescence, have been shown to contribute to PD^2^.

A detailed review of studies investigating aging in in vivo models of PD by this network highlight a complex and nuanced picture^2^. While some models show clear evidence of age-related disease progression, others do not. Phenotypes shared with PD were particularly evident in models where slow disease progression could be tracked and when animals were observed over extended periods of time^2^. These findings suggest that the influence of aging on PD is subtle, emerges gradually, and likely interacts synergistically with other contributing factors.

To advance our understanding of the role of aging in PD pathogenesis, the PD-AGE group emphasized the need for a more mechanistic approach. There was a strong consensus that crossing models of accelerated aging (reduced lifespan) or models with increased longevity (extended lifespan) with PD models would be a valuable strategy. This approach could clarify whether PD-like pathology can be either accelerated or slowed, respectively, shedding light on the systemic effects of aging underlying PD. To develop a manageable plan of work, the group decided to prioritize studies that involved crossing models of accelerated aging with PD models, with the rationale that they could yield novel experimental tools useful to test interventions within shorter timeframes, while simultaneously providing critical mechanistic insights.

## Selecting mouse models of Parkinson’s disease

Our group previously conducted an in-depth review of available mouse models of PD^2^, which informed the subsequent selection of representative models. The selection criteria prioritized models that capture the heterogeneity of the disease and represent distinct categories of PD, including early, mid, and late-onset forms as well as environmental influences (Table 1). Importantly, the focus was placed on models with incomplete penetrance and subtle PD phenotypes, excluding toxin-based models such as 6-OHDA (6-Hydroxydopamine) and MPTP (1-methyl-4-phenyl-1,2,3,6-tetrahydropyridine) or models with genetic deletion of mitochondrial genes in dopaminergic neurons, which, although useful to study the impact of dopaminergic neuron loss, do not model a physiological trigger of PD and develop a PD phenotype too rapidly. This deliberate approach aimed to assess potential synergistic effects when these models are crossed with models of accelerated aging. By using models with nuanced phenotypes, the group sought to determine whether such crossings amplify disease severity or accelerate the onset of PD-related characteristics. The group prioritized 5 mouse models: *Pink1*^*−/−*^ mice, models with PD-linked *Lrrk2* or *Gba* mutations, mice with intracranial injection of preformed fibril (PFF) as a model of α-synucleinopathy, and mice injected with paraquat as a toxin model.

**Table 1:** Summary of PD mouse models and characteristics considered for prioritization

Model	Relevance to PD in patients	PD phenotype	Potential disadvantages of crossing with accelerated aging models
*Pink1*^*−/−*^	Mutation in patients causing early onset	-Diminished DA release^60^ -Viral overexpression of α-synuclein in the SN resulted in DA degeneration and higher levels of pSer129 α-synuclein^15^	
*Parkin*^*−/−*^	Mutation in patients causing early onset	-Slight motor/behavioral deficits^61^, -Increased extracellular DA -Cross with A53T α-synuclein did not increase neurodegenration^61^	
*Gba*	-Associated with sporadic PD-More aggressive form of PD -most frequent mutation but low penetrance -Intermediate onset	-No overt PD phenotype^62^ -L444P mutation shows increased susceptibility to MPTP^25^	
*Lrrk2*	-G2019S mutation most frequent high-penetrance mutation in PD patients -Risk factor for familial PD -Late onset	-Lrrk2 overexpression or KI mutation shows subtle changes in dopamine metabolites and mitochondrial and lysosomal function^20^ -Excess *LRRK2* greatly accelerated the progression of neuropathological abnormalities in A53T α-*synuclein* transgenic mice^20,63^ 16/12/2025 22:20:00	
VPS35	-VPS35 p.D620N mutation present in <1% familial cases -late onset	-Age-related motor defects^64,65^ -Progressive degeneration of SN DA neurons -Increased DA release and widespread axonal damage -Tau-positive (hyperphosphorylated) pathology	
*Dj-1*^*−/−*^(C57Bl6)	-Familial PD	-Motor deficits^66^ -Loss of DA neurons	-Short lifespan -Early Severe disease (8 weeks old)
MitoPark	-Mechanistic model -Late onset, progressive	-Motoric deficits^67–69^ -Loss of DA neurons in SN -Expression of the mutation is restricted only to DA neurons^67^	-Short lifespan -Early disease onset
Overexpression Human WT α-synuclein (Thy-1, PDGF promoter)	Synucleinopathy Familial PD	-Widespread α-synuclein overexpression^70^ -Deficits in DA release^71^ -Early & progressive sensorimotor deficits^63,72^	Early motor deficits onset (2 months)
Overexpression Human WT, A30Pα-synuclein (BAC promoter)	Synucleinopathy Familial PD	-Widespread α-synuclein overexpression^73^ -Modest SN DA loss and gait disturbances -Deficits in DA release and DA neuron firing -WT and A30P α-synuclein exacerbates MPTP effects^73^	Requires higher number of animals than viral or PFF method due to breeding
Point mutations (A53T prp, A30P Thy-1)	Synucleinopathy Familial PD	-Motor deficits^74^ -Cognitive decline^75^ -Modest SN loss of DA neurons (A30P)^74^	Requires higher number of animals than viral or PFF method due to breeding
Truncated α-synuclein (N103)	Synucleinopathy Familial PD	-Early, formation of α-synuclein inclusions in neurons^76^. Progressive neurodegeneration in the SN and cortex. Age-dependent motor deficits	Requires higher number of animals than viral or PFF method due to breeding
Viral transfection of α-synuclein	Synucleinopathy Familial PD	-Extent of α-synucleinopathy is dependent on serotype^77–82^ -Progressive accumulation of α-synuclein aggregates in SN DA neurons -Progressive SN loss of DA neurons -Motor deficits	-Vector toxicity -Transduction efficiency can vary
Exogenous α-synuclein preparation - preformed fibrils (PFF), brain extracts	Synucleinopathy Familial PD	-Progressive Lewy body-like pathology^80,83,84^ -Modest and progressive DA neuronal loss -Motor deficits	-Challenging to generate pure preformed fibrils. -Different PFF strains cause different biological effects. -Validation of successful preparation is crucial
6-OHDA	Toxin	-Nigrostriatal damage^85^ -Motor deficits	Phenotype develops quickly. No sufficient time to detect changes
MPTP	Toxin	-DA neuron death^86,87^ -Motor deficits^88^	Phenotype develops quickly. No sufficient time to detect changes
Paraquat	Toxin, Epidemiological cause of PD	-Age and dose-dependent loss of DA neurons in the SN^89^ -Lower DA neuron loss compared to MPTP (30%)^90^ -Formation of Lewy body-like inclusions^91^ -Reduced locomotory activity^92–95^	
TCE	Toxin, Epidemiological cause of PD	-Loss of DA neurons in SN -α-synuclein accumulation^96^	Advance expertise for oral gavage
Rotenone	Toxin, epidemiological cause of PD (chronic exposure)	-DA cells loss in SNpc^97^ -Alpha-synuclein accumulation in SN (peripheral to central nervous system spread)^98–100^	Differences in solvent impact results

*6-OHDA* 6-hydroxydopamine, *MPTP* 1-methyl-4-phenyl-2,3-dihydropyridinium, *TCE* trichloroethylene, *SN* substantia nigra, *DA* dopaminergic, *WT* wild type.

The group strongly endorsed the Pten-induced kinase 1 (*Pink1*) knock-out (KO) mouse model, which involves the deletion or inactivation of the *Pink1* gene, mimicking the loss-of-function mutations seen in human patients. This model does not exhibit spontaneous overt neurodegeneration in the SNpc, even in aged animals, but develops mitochondrial dysfunction, subtle motor deficits (coordination and grip strength), and deficits in olfactory function and anxiety-like behaviors, commonly reported in the prodromal stages of PD. For these reasons, *Pink1*^*−/−*^ mice are considered a model of the early, preclinical stages of PD. In addition, this model has additional vulnerabilities related to PD, i.e., sensitivity to a second hit. Here, overexpression of α-synuclein results in greater synuclein pathology and DA neurodegeneration^15^, and increased axon terminal loss was shown to occur in the striatum in response to a gastrointestinal infection^16^. Together, this suggests that accelerated aging may result in similar effects. It was recognized that other genetic models of early-onset PD, such as *Park2*^*−/−*^ (*Parkin*^*-/-*^*)* mice, could have also been selected. However, the *Pink1*^−/−^ model has been more extensively characterized and, importantly, exhibits vulnerability to a second hit^17^.

Representing models of later age onset PD, the *Lrkk2* and glucocerebrosidase (*Gba*) models had moderate to strong levels of endorsement as they represent common mutations in patients who display a slow progression and lack of full penetrance. LRRK2 is a large, multidomain protein kinase that plays a critical role in familial and sporadic PD^18^. Mutations in LRRK2, particularly the G2019S mutation, are the most common genetic cause of autosomal dominant PD and are also implicated in sporadic cases. *Lrkk2*^-G2019S^ knock-in mice express the mutated protein at physiological levels, avoiding the potential overexpression artifacts obtained with transgenic models^19^. Although they fail to recapitulate the full spectrum of PD pathology, including not showing extensive dopaminergic neurodegeneration and α-synuclein aggregation, these mice exhibit motor impairments and synaptic dysfunction. In addition, excess Lrrk2 showed DA neurodegeneration following a cross with A53T α-*synuclein* transgenic mice^20^ and increased susceptibility to lipopolysaccharide (LPS)^21,22^, indicating vulnerability to a second hit.

Mutations in the *GBA* gene, which encodes the lysosomal enzyme glucocerebrosidase (GCase), are a significant genetic risk factor for PD^23^. Patients with a Gba mutation have one of the most aggressive forms of PD with an intermediate onset. These mutations disrupt lysosomal function and autophagy, contributing to the accumulation of α-synuclein and neuronal dysfunction. Knock-in models exist that introduce specific human *GBA* mutations (e.g., D409V, L444P) into the mouse genome, mimicking pathogenic variants found in PD patients^23^. The D409V GBA heterozygous mutant mouse does not show signs of dopaminergic neuronal loss or accumulation of α-synuclein, but it shows an increase in dopamine turnover in the striatum at 12 months^24^, considered one of the early signs of PD. The L444P GBA heterozygous mutant mice show progressive accumulation of total α-synuclein with age but no other sign of disease. However, they do show an increased vulnerability to the neurotoxin MPTP with increased loss of dopaminergic neurons in the SNpc compared to wild-type mice after MPTP treatment, and a greater reduction in TH-fiber density and behavioral deficits, which were exacerbated in GBA^*+/L444P*^ mice after MPTP treatment^25^, further suggesting vulnerability to a second hit.

The α-synuclein PFF model was prioritized as a model of synucleinopathy in sporadic PD because it induces abundant aggregates immunoreactive for serine 129-phosphorylated α-synuclein, which is a selective marker of human synucleinopathy, although other α-synuclein models (e.g., viral overexpression of wildtype or mutant forms of α-synuclein) were recognized to display similar (~30–50%) loss of dopaminergic neurons. On balance, this model was felt to have the least disadvantages when potentially combined with models of accelerated aging, provided there is good validation of the size (less than ~50 nm) of the PFF preparations required for efficient uptake and seeding. The model involves the injection of sonicated fragments of fibrils generated from recombinant purified mouse or human α-synuclein into the brain or peripheral tissues of mice, thereby seeding the conversion of endogenous α-synuclein into fibrils and inducing a cascade of α-synuclein aggregation and pathology^26^. This model mimics key features of sporadic PD, including the spread of α-synuclein pathology, intracellular inclusions resembling Lewy bodies, neuroinflammation, and partial dopaminergic neurodegeneration. The model also shows progressive motor impairments, including reduced coordination and bradykinesia, as well as non-motor symptoms characteristic of prodromal PD, such as olfactory deficits and anxiety-like behaviors.

Among the environmental models, there was strong agreement on selecting the widely used herbicide paraquat, considering its epidemiological relevance to PD and the induction of a phenotype that is intermediate in terms of severity relative to the very acute phenotype of the mitochondrial toxin MPTP or the very mild phenotype induced by rotenone, another mitochondrial toxin used as a pesticide^27,28^. Although more recently, low dose MPTP was shown to develop little or no signs of DAergic vulnerability at low dose, but did so in the context of viral infection as an additional stressor^29,30^, paraquat took priority for its epidemiological relevance. While preclinical studies on paraquat as a model for PD show variable results, they still generally indicate that paraquat can induce chronic neurodegeneration resembling early or preclinical stages of PD even in the absence of combined treatment with other agents such as Maneb. Although variability can occur, it is related to differences in dosing regimen and treatment duration.

## Selecting models of accelerated aging for Parkinson’s disease research

The group undertook a rigorous process to shortlist mouse models of accelerated aging to study their interplay with PD. The primary selection criteria focused on models exhibiting multiple features of accelerated aging, including the presence of multi-organ dysfunction, multiple hallmarks of aging, and reduced lifespan (see Tables 1–3 for a summary of these characteristics). Shortlisted models were Excision Repair Cross Complementation 1 knockout mice (*Ercc1*^*−/−*^*)*, Nuclear factor-kappa B p50 subunit knockout mice (*Nfkb1*^*−/−*^*)*, *Lamp2A* knockout mice (*Lamp2A*^*−/−*^), and *Klotho* knockout mice (Klotho^*−/−*^), all of which display systemic aging phenotypes. For the *Ercc1*^*−/−*^ and *Lamp2a*^*−/−*^ models, it was emphasized that a hypomorphic model is optimal where gene expression is attenuated rather than ablated and should be considered as a priority when available (i.e., for *Lamp2A*^*−/−*^ mice, heterozygosis is only possible in females since the *Lamp2* gene is X-linked).

**Table 2:** Mouse models of accelerated aging shortlisted for ranking and combining with PD mouse models, along with the description of their gene function

Mouse model	Disrupted gene
*Ercc1*^*−/−*^ *Ercc1*^*Δ/−*^	Excision Repair Cross Complementation 1 (*Ercc1*) is a necessary component in multiple DNA repair pathways. A decline in DNA repair has been associated with age and in PD^36^. The *Ercc1*^*−/Δ*^ hypomorphic mouse is engineered to produce a truncated version of the ERCC1 protein, lacking its C-terminal region, required to stabilize its binding partner XPF, the catalytic domain of the ERCC1-XPF DNA repair endonuclease. This truncation is achieved by introducing a premature stop codon into the *Ercc1* gene, resulting in a protein that is missing the last seven amino acids^36^. The truncation weakens but does not abolish XPF interaction, and ~5% of the normal levels of ERCC1-XPF are detected. Average lifespan depends on strains and modification: C57Bl6 *Ercc1*^*−/−*^ 3–4 weeks; *Ercc1*^*Δ/−*^ 6 months^36^.
*Nfkb1*^*−/−*^	Nuclear factor kappa (NF-kB) is a family of 5 hetero- or homo-dimeric transcription factors that regulate the expression of 200 genes. *Nfkb1* is a gene encoding p105, a precursor protein that is processed into p50, a key subunit of the NF-κB family of transcription factors. NF-κB plays a central role in regulating immune responses, inflammation, cell survival, and apoptosis^101–103^. Average lifespan is 12–18 months, depending on animal house^104,105^.
*Lamp2a*^−/−^	Chaperone-mediated autophagy is a selective form of autophagy that targets proteins bearing sequences with biochemical properties of the pentapeptide KFERQ for lysosomal degradation. KFERQ-like motifs are present in 50% of proteins, but may also be added through post-translational modification^106^. The motif is recognized by the HSC70 chaperone protein that subsequently docks onto the CMA lysosomal receptor, the lysosome-associated protein type 2A (LAMP2A), which mediates substrate translocation across the membrane. LAMP2A is the limiting component of CMA, and ablation of LAMP2A leads to CMA blockage^38^. While CMA can be stress-induced, it is also constitutively active in all cells^107^. CMA decreases with age^108^. Reduction in lifespan in mice with neuronal-specific deletion of LAMP2A but not upon systemic deletion^109^.
*Klotho*^*−/−*^	Klotho encodes a transmembrane protein that functions as a co-receptor for fibroblast growth factors (FGFs), particularly FGF23^39^. Average lifespan 8–9 weeks^39^.
*Ku70*^*−/−*^	Ku70 is a protein encoded by the XRCC6 gene and is a critical component of the Ku heterodimer, consisting of Ku70, Ku80. It is involved in non-homologous end joining (NHEJ) of double-strand breaks^110^. High mortality prior to weaning. For those who survive average lifespan is 37 weeks^110^.
*Csa*^*−/−*^	Cockayne syndrome group A protein, encoded by the ERCC8 gene. It plays a critical role in transcription-coupled nucleotide excision repair, responsible for excising DNA lesions caused by UV light and many other endogenous base-pair-disrupting, transcription-stalling genotoxic adducts^111^. No reduction in lifespan^112^.

**Table 3:** Hallmarks of aging identified in the six shortlisted accelerated aging mouse models

Ageing Hallmark	*Ercc1*^*−/−*^ *Ercc1*^*Δ/−*^	*Nfkb1*^*−/−*^	*Lamp2a*^*−/−*^	*Klotho*^*−/−*^	*Ku70*^*−/−*^	*CSA*^*−/−*^
Genomic instability	^36^	^101^	^113^	^114^	^115,116^	^117^
Telomere attrition		^104^		^114^		
Epigenetic alterations	^118,119^			^114^		
Loss of proteostasis	^120^		^38,121,122^	^114^		
Disabled macroautophagy	^123^			^114^		
Disrupted nutrient sensing	^124^		^113,125^	^114^		
Mitochondrial dysfunction	^120^		^121,126^	^114^	^127^	^128^
Cellular senescence	^36^^,^^129^	^103,104^	^130^	^114^		
Stem cell exhaustion	^62^	^104^	^121^	^114^		
Altered intercellular comm.	^131^	^101,103^				
Chronic inflammation	^132^	^101,104,133^	^113,134,135^	^114,136^	^137^	^138^
Dysbiosis^a^	^139^					

References provided experimental evidence of each hallmark, when available.

^a^While dysbiosis was not measured in *Ercc1*^*Δ/−*^ mice, probiotic treatment was shown to improve mucosal lining and reduce inflammation.

Additionally, the group explored the potential of other mouse models carrying alterations in DNA damage repair processes that are essential for neurons, such as *Ku70* mutants for non-homologous end-joining and Cockayne syndrome group A (*Csa* or *Ercc8*) mutants for nucleotide excision repair. *Ku70* and *Ercc8* have not been directly associated with PD, even though some evidence indicates an interaction between ERCC8 and Parkin^31^. Moreover, both KU70 and CSA have been implicated in other neurological disorders^32–35^. Finally, *Ku70* and *Ercc8* mouse mutants display a mild phenotype, are amenable to breeding with mouse strains harboring PD gene mutations, and were therefore highlighted as promising candidates for bridging the gap between aging mechanisms and PD pathogenesis. The telomerase reverse transcriptase knock-out model (*Tert*^−/−^ and *Terc*^*−/−*^), characterized by signs of accelerated aging across multiple tissues and shorter lifespan, was also considered. However, challenges in breeding and uncertainties surrounding the relevance of telomere shortening to brain aging raised concerns about its suitability for PD research.

Models related to mitochondrial dysfunction, such as the polymerase gamma gene mutator mouse model *(Polg* D257A/D257A*)*, mitochondrial transcription factor A knock-out mice (*Tfam*^−/−^) and modified NADH:ubiquinone oxidoreductase core subunit S2 (*Ndufs2*^*−/−*^), were also evaluated due to their common relevance to hallmarks of aging and PD but ultimately not endorsed. The limitations of these models included the limited evidence that they were models of accelerated aging (*Tfam*^*−/−*^ and *Ndufs2*^*−/−*^), the segmental (tissue-specific) nature of their defects, and the variability in the reproducibility of phenotypes (*Polg*^D257A/D257A^). These constraints made them less suitable for comprehensive studies of systemic aging in the context of PD.

To refine the selection process, the models were ranked based on three criteria: (1) the presence of neuronal vulnerability or loss, (2) evidence of multiple hallmarks of aging, and (3) the occurrence of multi-organ dysfunction (Tables 2–4). This systematic evaluation ensures that the selected models not only represent accelerated aging but also provide relevance to PD, facilitating a more integrative understanding of the disease. All models show reduced lifespan, although in the case of *Lamp2a*^*−/−*^, this reduction is more evident when the gene is selectively deleted in neurons than in the systemically deleted model^36–39^. *Ercc1*^*−/Δ*^, *Nfkb1*^−/−^, *Lamp2a*^*−/−*^, and *Klotho*^*−/−*^ show dysregulation of multiple hallmarks of aging and dysfunction in multiple tissues. *Ercc1*^*−/Δ*^ and *Lamp2a*^*−/−*^ show abnormalities in dopaminergic neurons in the SNpc and accumulation of α-synuclein. Analysis of dopaminergic neurons was not available for the *Klotho*^*−/−*^ mouse model. However, *Klotho*^+/−^ mice showed a significant decrease in the number of TH-positive neurons in the SNpc compared to that in WT mice^40^, changes in morphology, and a significant decrease in striatal dopamine levels. *Nfkb1*^*−/−*^ did not show dopaminergic neuron loss per se, but the number of TH+ neurons in the SNpc was reduced more extensively in these mice compared to controls when the mice were exposed to MPTP, suggesting increased neuronal vulnerability^41^. We were unable to find information on neuronal vulnerability for the *Ku70*^*−/−*^ and *Csa*^*−/−*^ mouse models.

**Table 4:** Presence of age-related decline in organ function for the six shortlisted models of accelerated aging

Mouse model	Neurologic abnorm.	CVD	Kyphosis	Sarcopenia	Bone loss	Organ atrophy	Sensory loss	↓body weight/fat	↓GI function	Emphysema/Lung	↑Cancer	↓Immune function	Hair loss	↓Insulin sensitivity
*Ercc1*^*−/Δ*^	^120,140,141^	^142,143^^b^	^144,145^	^144,146^	^147^	^144,145^	^144,148,129^	^149^	^139,150^	^143^^b^	^151^^b^	^131^^b^		^152^^b^
*Nfkb1*^*−/−*^	^133^	^101,153^		^101,154^	^103,147^			^104^	^155^	^105^	^102,155^	^37,104^		
*Lamp2a*^*−/−*^	^38,122,156,157^	^113,135^			^158^	^121^		^159^				^160^		^113,159^
*Klotho*^*−/−*^	^39^	^39,161^	^162^	^114^	^162^	^162^		^162^		^162^		^39,161^	^162^	^162^
*Ku70*^*−/−*^	^32–35^		^115^						^110,137^		^115^^a^	^115^^,163^	^115^	
*Csa*^*−/−*^	^138^						^112,117^	^138^						

References provide experimental evidence of each hallmark, when available. Neurological abnormalities include dystonia, tremors, ataxia, decreased neuronal function, reduced plasticity, cognitive impairment neurodegeneration and increased blood-brain barrier permeability (*Ercc1*^*−/Δ*^); enhanced neuroinflammation and cognitive decline (*Nfkb1*^*−/−*^); gradual cognitive and motor deficiencies, gait disturbance and hypokinesis reminiscent of PD (*Klotho*^*−/−*^); brain inflammation (Cockayne Syndrome (*Csa*^*−/−*^)). Organ atrophy includes skin, intestine, gonads, thymus, and/or bone marrow.

*CVD* cardiovascular disease.

^a^Indicates enhanced cancer incidence in Ku70^−/−^ dependent on mouse background.

^b^Indicates data derived from tissue-specific deletion of *Ercc1*.

Based on this information, the group prioritized Excision Repair Cross Complementation 1 (*Ercc1*^*−/Δ*^) and Nuclear factor-kappa B (*Nfkb1*^−/−^) deficient models for crossing with PD models due to the richness of information available. *Lamp2a*^*−/−*^ and *Klotho*^*−/−*^ deficient mice could be considered as a close second. However, it was felt that *Ku70*^*−/−*^ and *Csa*^*−/−*^ are not sufficiently characterized at this time (Table 5).

**Table 5:** Summary table highlighting studies supporting the presence of DAergic neuronal cell vulnerability in the *Ercc1*^*−/Δ*^, *Lamp2a*^*−/−*^*, Nfkb1*^*−/−*^, and *Klotho*^*−/−*^ models

Mouse model	Evidence for Dopaminergic (DAergic) neuronal vulnerability
*Ercc1*^*Δ/−*^	• ERCC1 is necessary for DAergic neuronal function^120^ • Knockout specific to DA neurons (*Ercc1*^−/−fl/fl^DAT-Cre^+^) shows decrease in TH immunostaining in the SNpc and striatum at 26 weeks, with further decrease at 52 weeks as compared to control. • *Ercc1*^*Δ/−*^ shows ο Decreased innervation of DAergic neurons in the striatum, but not the SNpc, at 20 weeks of age. ο Early increases in relative TH staining in the striatum hypothesized to be due to compensatory up-regulation to replace losses in dopamine occurring prior to frank cell loss. ο Increased phosphorylation of α-synuclein (pSer129), an indicator of α-synuclein aggregation, but lack of detectable aggregates.
*Nfkb1*^*−/−*^	• *Nfkb1*^*−/−*^ mice showed signs of brain aging, neuroinflammation, and cognitive decline that were rescued by Ibuprofen treatment^133^, decrease in DCX-positive neurons in the DG • *Nfkb1*^−/−^ mice exhibited exacerbated microglial activation and dopaminergic neuron loss after MPTP treatment^41^.
*Lamp2a*^*−/−*^	• WT α-synuclein and LRRK2 can be degraded by CMA^164,165^ • A53T mutation^164^ and chemical modifications by dopamine^166^, as well as mutations in LRRK2^165^ and GBA^167^, all halt degradation via CMA, leading to collapse of the neuronal metastable proteome^38^ • Knock out of neuronal *Lamp2a* increases α-synuclein aggregates^38,157^ • Brain from *Lamp2a*^−/−^ mice recapitulate 42% of the changes in the proteome observed in GBA-PD patients’ brains^167^
*Klotho*^*−/−*^	• Klotho knockdown in 9.5-week mice^40^ resulted in: ο Significant decrease in the number of TH-positive neurons in SNpc compared to WT mice. ο Much smaller TH-positive neurons in SNpc of KIotho mice than those in WT. ο Reduced TH staining in the VTA but not in the LC. ο Significant decrease in striatal dopamine levels, specific for the dopaminergic system. ο Vitamin D restrictive diet (KI knock-down mice have high levels of Vit D) increased number of TH+ SNpc neurons and restored dopamine levels similar to those of WT. α-synuclein deposits were not assessed.

No evidence of DAergic neuronal vulnerability was found in *Ku70*^*−/−*^ and *Csa*^*−/−*^ models.

*TH* tyrosine hydroxylase, *SNpc* substantia nigra pars compacta, *pSer129* α-synuclein at Serine 129, *DCX* doublecortin, *DG* dendate gyrus, *MPTP* 1-methyl-4-phenyl-1,2,3,6-tetrahydropyridine, *VTA* ventral tegmental area, *aKl-F* Kl fragment, *Vit D* vitamin D, *XP* xeroderma pigmentosum, *CS* Cockayne Syndrome, *DMV* vagus nerve, *OB* olfactory bulb, *LC* locus coeruleus, *Snca* alpha-synuclein, *WT* wild type, *Kl* Klotho, *BBB* blood-brain barrier.

## Optimizing experimental design for characterizing mouse models of PD and accelerated aging

To establish an experimental framework for characterizing aging PD mice, participants were provided with a comprehensive list of tests through a questionnaire, followed by group discussions. Tests were categorized into Tier 1, prioritized for all new crosses, and Tier 2 (Table 6), reserved for mouse lines showing accelerated or enhanced PD phenotypes based on Tier 1 tests. This approach was taken in order to balance feasibility with scientific depth. In the first instance, gold-standard histological endpoints were selected as they provide the most direct and well-validated measures of PD pathology. If these analyses reveal a phenotype, the experiments will proceed to Tier 2 assays. These second-stage tests were selected because they capture domains of PD that extend beyond nigrostriatal degeneration but are more resource-intensive or technically challenging in aging cohorts. This tiered approach was selected to ensure detection of interesting phenotypes, keeping in mind a number of tests that are manageable on the same cohort, are cost- and time-effective, and are observant of the welfare of older animals. Selection criteria for Tier 1 tests included invasiveness, resource intensity, timing, and the feasibility of longitudinal vs. cross-sectional assessments.

**Table 6:**
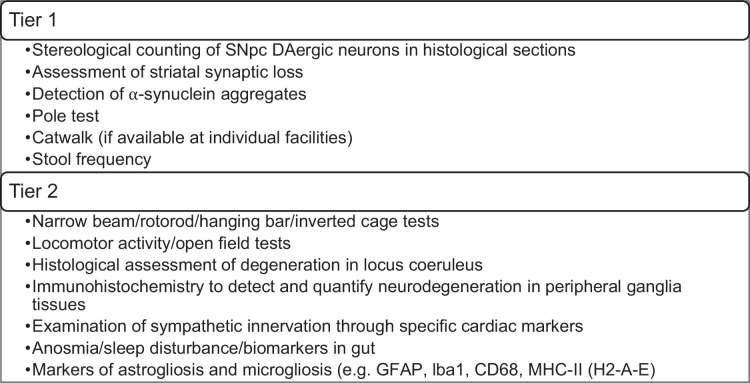
Tier 1 and Tier 2 priority tests for assessing PD characteristics in PD-aging mixed mouse models

*SNpc* substantia nigra pars compacta.

The consensus for Tier 1 tests comprised histological analysis with the counting of dopaminergic neuronal cell loss in the SNpc, the quantification of loss of striatal dopaminergic innervation, and the quantification of α-synuclein aggregates. Two behavioral tests were selected: the pole test (sensitive to dopaminergic neuron loss) and the catwalk gait analysis (if available) (Table 6). Additionally, stool frequency was included as a low-resource metric indicative of early PD symptoms. These 3 histological tests were chosen because they assess the core pathological hallmarks of PD. Specifically, stereological counting of dopaminergic neurons in the SNpc is the primary outcome as it provides a direct and quantitative measure of the cardinal pathology underlying motor symptoms. This protocol has been standardized by the group (see 10.5281/zenodo.16685628). Further, quantification of striatal dopaminergic innervation loss complements this by capturing the integrity of nigrostriatal projections, which closely correlates with motor deficits and disease severity. Additionally, loss of striatal DA axon innervation can be quantified in multiple ways, the most common approach being quantification of TH, DAT, or VMAT2 immunohistochemical staining in coronal sections of the striatum. A basic protocol for this can be found here^42^.

In addressing the key molecular signature of PD, measurement of α-synuclein aggregates would allow the assessment of whether aging or genetic background modifies proteinopathy. Quantification of α-synuclein aggregates is best accomplished by immunostaining coronal brain sections using antibodies for serine 129-phosphorylated α-synuclein, then analyzing images with ImageJ or similar software to quantify the number or density of immunoreactive aggregates in each region of interest^43^. Pre-treatment with proteinase K can be used to quantify only dense, proteinase K-resistant protein aggregates, although this is not necessary because most antibodies against serine 129-phosphorylated α-synuclein selectively label neuropathological protein aggregates and do not label the more abundant monomeric or non-aggregated α-synuclein. For comparison of antibodies, see ref. ^44^.

For behavioral assessments, the pole test was selected because it is simple, non-invasive, reproducible, and requires no training, which makes it well-suited for longitudinal studies in aging cohorts. It specifically captures bradykinesia and motor coordination, which are cardinal motor symptoms of PD^45^. It is also more sensitive than open-field or rotarod tests for detecting subtle motor deficits in PD models, particularly in early or mild phenotypes. Specifically, rotarod performance depends heavily on cerebellar and cortical motor systems and requires training. Mice with dopaminergic deficits may retain near-normal performance, relying primarily on coordination and balance, provided cerebellar and vestibular systems remain intact^46^. An open field can reflect hypokinesia/akinesia, but exploration can also be strongly influenced by anxiety^47^. The catwalk was also selected when assessment is possible because it can detect subtle changes in locomotion that are difficult to capture by eye. It also has high translational value for motor symptoms beyond bradykinesia, as PD patients show characteristic gait abnormalities (shorter steps, reduced stride length, altered rhythm). However, it was ultimately considered optional because it requires specialized knowledge both in gait analysis and the handling of older, frailer animals. It was also noted that the pole test and the catwalk gait test require a significant lesion to detect a phenotype^48^, thus the absence of behavioral changes should not preclude histological analysis. Decisions to proceed with further tests should be based on the outcomes of these initial assessments, with tissue storage for *omics* analyses prioritized for future studies.

Tier 2 tests included the examination of further measures of locomotion, PD biomarkers in gut tissues or fecal samples, the quantification of sympathetic innervation through cardiac biomarkers, and the assessments of sleep disturbances, measures that are relevant to PD but require more resources (Table 6). Here, motor coordination and strength are evaluated with narrow beam, rotarod, hanging bar, and inverted cage tests, which complement the pole test by detecting subtle deficits in balance, bradykinesia, and endurance^45,49–51^. Spontaneous activity is assessed in the open field, where reduced exploration may reflect diminished dopaminergic function^52^. Because early noradrenergic degeneration is a recognized feature of PD^53^, we propose to examine the locus coeruleus histologically, while immunohistochemistry of peripheral ganglia captures pathology in the autonomic nervous system, another important contributor to disease. In addition, we assess cardiac sympathetic innervation, reflecting the autonomic failure that is both common and clinically significant in PD^54,55^. Non-motor domains will be addressed through the evaluation of sleep and olfactory function, which often present as prodromal symptoms in patients^56,57^. Finally, gut biomarkers will be examined to detect α-synuclein pathology and physiological changes in the enteric nervous system, a compartment increasingly implicated in PD initiation and progression^58,59^. Together, these measures will provide a multidimensional view of phenotypes and increase the translational relevance of our models.

For experimental timing, given the widely different lifespans of the accelerated aging models (e.g., *Ercc1*^*Δ/−*^ 24–30 weeks and *Nfkb1*^*−/−*^ 12–18 months), the group agreed that pilot studies should be conducted at multiple time points and tailored to the lifespan and PD characteristics of the specific models. Times will be centered around the period just before increased mortality is observed and when the accelerated model of aging shows increased hallmarks of aging, such as senescence, inflammation, and multiple tissue deficits. After behavioral tests have been completed, mice should be anaesthetized and intracardiac paraformaldehyde perfusion performed, followed by histological analysis, including unbiased stereological counting of dopaminergic neurons in the SNpc and quantifying the density of dopaminergic innervation in the striatum using tyrosine hydroxylase immunohistochemistry to estimate axon terminal loss. Repeated pole testing on the same animal was discouraged due to the potential for learning effects, which could compromise reliability. The results of these studies are crucial to the design of the experiments for Tier 2 endpoints. We posit that this structured, phased approach balances accurate characterization with resource efficiency, optimizing the evaluation of these complex models.

## Concluding remarks

In conclusion, aging represents a critical yet understudied risk factor for PD, warranting a concerted effort to bridge gaps in understanding the role of aging biology in disease onset and progression. The complexity and heterogeneity of PD models, combined with the lengthy nature of aging studies, present challenges that require substantial resources and innovative approaches. This collaborative roadmap, developed by experts in aging and in PD research, aims to standardize methodologies, foster cooperation, and optimize resource utilization. The initial focus on combining selected PD models (e.g., *Pink1*^*−/−*^, mutations in *Gba and Lrrk2 genes*, α-synuclein PFF injection, and paraquat exposure) with accelerated aging models (e.g., *Ercc1*^−/Δ^ and/or *Nfkb1*^−/−^) offers a promising strategy to accelerate the discovery of novel insights into the interplay between aging and PD pathology.

This approach not only sets the stage for identifying key mechanistic links but also lays the groundwork for establishing preclinical PD models that incorporate aging as a central element of disease pathogenesis. By emphasizing tiered experimental designs, the proposed roadmap ensures a balance between depth of analysis and feasibility/economy. The proposed approach is anticipated to advance therapeutic testing and ultimately contribute to the successful translation of strategies to delay or mitigate PD in patients. The collective vision laid out here is a future where aging-informed research propels the development of more effective interventions, transforming the understanding and treatment of PD.

## Data Availability

No datasets were generated or analyzed during the current study.
